# *SKCG-1*: a new candidate growth regulatory gene at chromosome 11q23.2 in human sporadic Wilms tumours

**DOI:** 10.1038/sj.bjc.6603090

**Published:** 2006-04-11

**Authors:** K P Singh, D Roy

**Affiliations:** 1 Department of Biology, Texas Southern University, Houston, TX 77004, USA; 2 Department of Environmental and Occupational Health, Stempel School of Public Health, Florida International University, Miami, FL 33199, USA

**Keywords:** novel gene, Wilms tumour, growth regulatory gene, chromosome 11q23.2

## Abstract

Using arbitrary primed-PCR (AP-PCR), we have identified a novel genetic alteration located at chromosome 11q23.2 and this genetic alteration was common in 38% of the human Wilms tumour samples analysed. Further characterisation by cloning and sequencing of this genomic region revealed that it represents a part of an uncharacterised gene. We have named this gene as Sporadic Kidney Cancer Gene-1 (*SKCG-1*). Using fluorescence *in situ* hybridisation (FISH) approach, we established its localisation on the chromosome 11q23.2. Northern analysis revealed the transcript size of *SKCG-1* of 2.09 kb and this was further confirmed by full-length cDNA sequence. Sequence analysis revealed an active translation start site (ATG sequence), a polyadenylation signal sequence (AATAAA), and an open reading frame (ORF) encoding a peptide of 124 amino acids in the cDNA sequence of *SKCG-1*. Analysis of genomic sequence of *SKCG-1* revealed a promoter region containing TATA box located at −13 bp upstream of transcription start site. The AP-PCR, SCAR, and Southern blot analyses indicated genomic loss of *SKCG-1* in Wilms tumours. The transcript of *SKCG-1* was abundantly present in brain, kidney, liver, testis, salivary gland, foetal brain, foetal liver, whereas relatively lower expression in heart, stomach, prostate and no expression in spleen, colon, lung, small intestine, muscle, adrenal gland, uterus, skin, PBL, and bone marrow was detected. The expression of this gene transcript was either very less or undetectable in Wilms and breast tumours compared to their matched uninvolved tissues. Inhibition of *SKCG-1* by siRNA resulted in increased cell proliferation of kidney epithelial cells. Based on the presence of two transmembrane regions in its peptide, *SKCG-1* has been predicted as a transmembrane protein. Thus, the findings of this study revealed (i) *SKCG-1*, a new gene located at 11q23.2 and harbouring genetic alteration in Wilms tumours, (ii) the presence of *SKCG-1* gene transcripts in various human normal tissues and its lower expression or absence in Wilms and breast tumours indicate that it may be associated with tumour growth suppressor activity, (iii) the presence of an open reading frame in the cDNA sequence of *SKCG-1* indicates that it has potential to encode a protein, (iv) increased cell growth by silencing this gene in HEK293 cells further supports a potential role of this gene in growth of kidney epithelial cells. Our findings suggest that *SKCG-1* may have a tumour suppressor role, and implicate genetic alteration in this gene as a potential oncogenic pathway and therapeutic target in kidney and breast cancer.

Altered expression of those genes that control cell proliferation and differentiation is a common end result of genetic alterations that occur in human tumours and is a crucial step in tumorigenesis. Identification and characterisation of all possible genetic alterations that predispose to cancer are of utmost importance to understand the development of cancer. In this study, by using arbitrary primed-PCR (AP-PCR) assay, we identified a new gene, *SKCG-1*, harbouring genetic alteration in 38% of the human sporadic Wilms tumour samples analysed. Wilms tumour or Nephroblastoma is an embryonal malignancy of the kidney, affecting about 1 in 10 000 children (Matsunaga, 1981). It comprises about 6% of all childhood cancers and is a major paediatric cancer burden at the global level (Volkher *et al*, 2001). The important discoveries made in the past several years regarding molecular events underlying Wilms tumorigenesis have indicated the involvement of two tumour suppressor genes namely *WT1* and *WT2* located at 11p13 and 11p15, respectively (Mannens *et al*, 1988; Koufos *et al*, 1989; Call *et al*, 1990; Gessler *et al*, 1990). Besides these two genes, many other mutational events, such as allelic loss of chromosome 16q (Maw *et al*, 1992), loss of heterozygosity at 11q23.3-q-ter region (Radice *et al*, 1995), and high rate of *P53* mutations located at 17p13 (Bardeesy *et al*, 1994) in Wilms tumour indicate the involvement of these genes/loci with Wilms tumour formation. Association of a particular genetic abnormality with specific histopathology of Wilms tumour indicates that WT development involves several, probably alternative, genetic pathways, but there is still an incomplete picture as to the identity of most of these genes, or the mechanisms by which they are controlled (Grundy *et al*, 1994; Hing *et al*, 2001). Thus, a complete description of molecular defects in individual WTs is highly warranted.

Using AP-PCR method, we have recently identified a novel mutation located at 17q11.2 in 80% of the sporadic breast tumour samples analysed (Singh and Roy, 2001). Here we report the identification and characterisation of a new gene, *SKCG-1*, which is altered in human Wilms and breast tumours. We have cloned the full-length cDNA sequence of *SKCG-1*, established its chromosomal localisation, determined its structural analysis and coding potential, analysed its expression in various normal human tissues, including Wilms and breast tumours and their corresponding uninvolved tissues, and tested its function in cell growth by silencing it.

## MATERIALS AND METHODS

### Chemicals

The AmpliTaq DNA polymerase (Stoffel fragment), dNTPs and mineral oil were purchased from PE Applied Biosystems (Foster City, CA, USA). A set of 20 OPA series oligonucleotide random ten-mer primers was purchased from Operon technologies (Alameda, CA, USA). The DIG high prime DNA labelling and detection kit was obtained from Roche Diagnostics (Indianapolis, IN, USA).

### DNA and RNA extraction

Thirteen human Wilms tumour tissues (six Caucasian American cases, six African-American cases, and one Asian), out of which 10 with matching tumour-normal pair and three tumours without matching normal as well as seven breast tumours with matching normal tissues were obtained through NCI Tissue Procurement Facility, Comprehensive Cancer Center (University of Alabama at Birmingham, USA). Human embryonic kidney epithelial cell line, HEK-293, was obtained from ATCC and propagated in DMEM/F12 growth medium with 10% FBS. Genomic DNAs were isolated from tumour and corresponding normal frozen tissues following the methods as described previously by us (Singh and Roy, 1999). Total RNA was isolated from the kidney and breast tumours, their corresponding uninvolved tissues, as well as from *in vitro* grown embryonic kidney epithelial cells (HEK-293) following the Trizol method (Invitrogen, Carlsbad, CA, USA). The DNA and RNA were quantified spectrophotometrically, and purity as well as integrity was checked by ethidium bromide staining after resolving on 1.5% agarose gel (for DNA) and on 1% agarose gel in 1 × formaldehyde buffer (for RNA).

### AP-PCR

AP-PCR was performed following the method as described previously by us (Singh and Roy, 2001). Amplifications were carried out in 25 *μ*l of reaction mixture containing 100 *μ*M each of dATP, dCTP, dGTP, dTTP; 2.5 mM MgCl_2_; 2.5 *μ*l of 10 × enzyme assay buffer, 1.5 unit of Taq DNA polymerase (stoffel) and 75 ng of genomic DNA. From a total of 15 primers used, the OPA14 (5′-TCTGTGCTGG-3′) was the only primer that revealed genetic alteration in the AP-PCR profile of Wilms tumour DNA. DNA amplification was performed in a Perkin-Elmer Cetus DNA thermal cycler programmed for 45 cycles: first cycle (3.5 min at 92°C, 1 min at 34°C, 2 min at 72°C), next 44 cycles (1 min at 92°C, 1 min at 34°C, 2 min at 72°C), followed by a final extension cycle of 15 min at 72°C. AP-PCR products were separated by electrophoresis in denaturing (4.0% acrylamide and 50% urea) sequencing gel and visualised by silver staining method (Singh and Roy, 1999). Each experiment was repeated three to four times.

### Cloning and sequencing of AP-PCR amplified product

AP-PCR product of 475 bp amplified by OPA14 (5′-TCTGTGCTGG-3′) primer was cloned and sequenced for further characterisation. This band in the AP-PCR fingerprint of uninvolved kidney DNA was excised from the gel, and DNA was eluted. The eluted DNA was reamplified with the same AP-PCR primer as before and using the same concentrations of reaction mixture constituents and PCR cycle conditions as described above. The PCR product was analysed on agarose gel to confirm its size and purity. The reamplified 475 bp DNA was cloned using TA cloning kit (Invitrogen, Carlsbad, CA, USA). The presence of an appropriate insert size was determined by restriction analysis of recombinant plasmid DNA, and it was further confirmed by hybridising it with pure 475 bp AP-PCR amplified DNA as a probe. The insert was sequenced with automated DNA sequencer through DNA sequencing Core Facility, Comprehensive Cancer Center, UAB. Sequences obtained from several clones were compared with known sequences in the GenBank database using the BLASTn and BLASTx program.

### Southern blot analysis

Total DNA was digested individually with restriction enzymes (*BamH1*, *Sma1*) according to the manufacturer's instructions and fractioned by 0.85% agarose gel electrophoresis (AGE) in TAE buffer (Maniatis *et al*, 1982). The separated DNA was transferred to nylon membrane (Hybond N^+^, Amersham Pharmacia Biotech Ltd.) by Southern blotting. The membrane was probed with [*α*-^32^P]-dCTP random primed-labelled 475 bp AP-PCR amplified fragment (cloned) as *SKCG-1* probe and the signals were detected by autoradiography of X-ray films. The same membrane was stripped off the *SKCG-1* probe and then rehybridised with *GAPDH* probe.

### Northern blot analysis

Total RNA (5, 10, 20 *μ*g) isolated from HEK 293 cells was fractionated by electrophoresis in 1.0% agarose gel containing formaldehyde and transferred to Hybond-N^+^ (Amersham) by capillary blotting. Membranes were hybridised with non-radioactive PCR-generated-DIG-labelled *SKCG-1* or *GAPDH* (glyceraldehydes-3-phosphate dehydrogenase) gene-specific probes and washed according to protocols provided by the manufacturer of DIG high prime DNA labelling and detection kit (Roche).

### Isolation and sequencing of full-length SKCG-1 cDNA

The full-length *SKCG-1* cDNA sequence was obtained by EST sequencing and Rapid Amplification of cDNA ends (RACE) method as described previously (Frohman, 1993). A 1.37 kb sequence of *SKCG-1* gene was obtained by sequencing of an EST clone (GenBank accession number AA935177) showing 100% similarity with 475 bp sequence. The remaining (0.720 kb) sequence was obtained by 5′ and 3′RACE. 5′ and 3′ RACE-PCRs were carried out with total RNA obtained from normal human kidney and using the GeneRacer kit (Invitrogen). PCR reactions were carried out as specified by the manufacturer of GeneRacer kit (Invitrogen). To obtain 5′-ends, initial PCR was performed with gene-specific primer (5′-GCTGCGCTGTGGGTATGTAAGATGTT-3′) followed by a nested PCR with nested primer (5′-GGATACACAGGGACTGCTTTG-3′) to increase the gene specificity of PCR product. Similarly, 3′-ends sequence was obtained by 3′RACE using gene-specific primer (5′-GGAGGCACCACTTGGTAACA-3′) and nested PCR with primers (5′-CCTGAGTGTCTCTGCCGTGT-3′). These GSPs were designed based on sequence data obtained from 1.37 kb EST sequence. Final full-length cDNA sequence of *SKCG-1*, thus obtained, was deposited to NCBI GenBank and the assigned accession number is *AY662656*.

### Bioinformatic analysis of SKCG-1

The sequence homology search and ORF analysis was performed by using software available at NCBI Website (www.ncbi.nlm.nih.gov). The prediction of potential methylation sites (CpG sites) in the promoter region sequence of *SKCG-1* was analysed by computer-based free software (www.ebi.ac.uk/servicestmp). The type of protein (soluble or membrane) and its cellular localisation was predicted by using SOSUI software system (http://sosui.proteome.bio.tuat.ac.jp). The detail structure of *SKCG-1* peptide, location of transmembrane region in the peptide, its hydropathy profile and helical wheel diagram is available at the EMBL bioinformatic harvester website (http://harvester.embl.de/harvester/Q51SC8).

### Reverse transcriptase-polymerase chain reaction (RT–PCR)

The transcript level of *SKCG-1* was measured by semiquantitative PCR. Oligo dT-primed first strand cDNA was synthesised from DnaseI-treated total RNA (2 *μ*g) using Superscript II reverse transcriptase (Invitrogen). The PCR amplification was performed in a Perkin-Elmer Cetus DNA thermal cycler programmed for first cycle of 94°C for 3 min, followed by 35 cycles 1 min of denaturation at 94°C, 1 min of annealing at 59°C, 1 min of extension at 72°C. The PCR was completed by a final extension cycle at 72°C for 10 min. PCR reactions with *GAPDH*-specific primers were also performed as control. *SKCG-1* gene-specific PCR fragment of 300 bp was amplified by forward (5′-GATAGGGAAGCCAAAGACAC-3′) and reverse (5′-CCAGAGCAGGAGGATAATAAA-3′) primers. Similarly, a 367 bp fragment of housekeeping gene, *GAPDH* was amplified by forward (5′-GTCGCTGTTGAAGTCAGAGGA-3′) and reverse (5′-TTCATGACAACTTTGGTATCG-3′) primers. Samples were analysed by electrophoresis on 1.5% agarose gels.

### Tissue distribution of SKCG-1 gene expression

The expression of *SKCG-1* gene among the various tissues was analysed by PCR using tissue-specific cDNA panel (OriGene Technologies). The primers and the PCR conditions were the same as mentioned in the RT–PCR protocol. Reactions with *β*-actin-specific primers were used as a control according to the manufacturer's instructions.

### Chromosomal localisation of the SKCG-1 gene by fluorescence *in situ* hybridisation

Chromosomal localisation of *SKCG-1* gene was performed by FISH following the method of Stokke *et al* (1995). Human BAC clone genomic library was screened using original 475 bp AP-PCR fragments as probe. Sequencing of the positive BAC clones using the primer designed from 475 bp region revealed a clone with entire 475 bp sequences. Plasmid DNA was purified from this clone and was labelled with digoxigenin dUTP by nick translation. Labelled probe was combined with sheared human DNA and hybridised to normal metaphase chromosomes derived from phytohaemagglutinin-stimulated peripheral blood lymphocytes in a solution containing 50% formamide, 10% dextran sulfate and 2 × SSC. Specific hybridisation signal were detected by incubating the hybridised slides in fluoresceinated anti-digoxigenin antibodies followed by counter staining with 4-6-diamidino-2-phenylindole (DAPI) for one-colour experiments. Probe detection for two-colour experiments was accomplished by incubating the slides in fluoresceinated antidigoxigenin antibodies and Texas red followed by counterstaining with DAPI. Ideograms and estimates of distance of *SKCG-1* gene locus from the centromere were calculated following the method as described by Francke (1994).

### SKCG-1 gene transcript silencing by siRNA

Silencing of *SKCG-1* was achieved by using the Dicer siRNA generation kit (Gene Therapy Systems, Inc., CA, USA) and following the manufacturer instructions. Briefly, double-stranded RNA (dsRNA) template was generated by T7 RNA polymerase-mediated *in vitro* transcription from 500 bp double-stranded target genomic region of *SKCG-1*. Double-stranded RNA was digested with the recombinant dicer enzyme to generate 22 bp siRNA. Overnight grown 50–70% confluent adherent HEK293 cells were transfected with siRNA following the Lipofectamine method. To determine the extent of gene silencing, total RNA was isolated from siRNA transfected and untransfected (as control) HEK293 cells after 48 h of post-transfection. RT–PCR was performed using the total RNA and transcript level of *SKCG-1* was determined following the method as described above in RT–PCR section. Cell growth was measured by counting the cells before isolating the RNA from these cells.

## RESULTS

### Identification of mutation in human Wilms tumour samples

**Figure 1:**
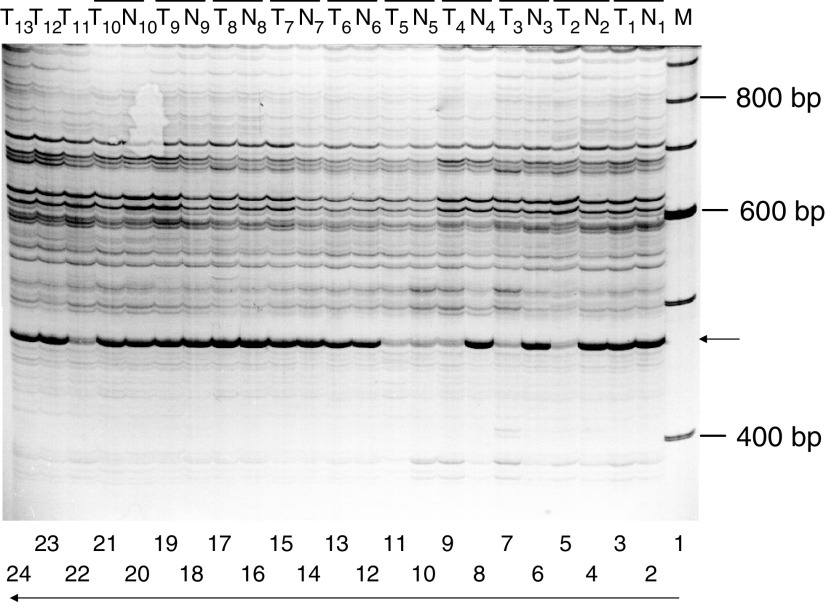
Representative AP–PCR fingerprints generated by primer OPA14 (5′-TCTGTGCTGG-3′) from Wilms tumours (T_1_–T_13_) and corresponding uninvolved normal (N_1_–N_13_) kidney tissue DNA. Arrow indicates the 475 bp AP–PCR amplified product with reduced intensity in 5 (T_2_, T_3_, T_4_, T_5_, and T_11_ of the Wilms tumour DNA samples as compared to the corresponding normal kidney tissue DNA from same patient.

To screen the mutations at genome-wide level, we compared the genomic fingerprint patterns of human Wilms tumours *vs* matched nonmalignant (uninvolved) tissues by AP-PCR analysis. Out of 15 different random primers used, one primer (OPA14) revealed multifold decreased intensity of 475 bp AP-PCR amplified DNA fragment in 38% (5 of 13) of the tumour samples as compared to uninvolved kidney tissues from the same individuals (Figure 1). Out of these five patients, one was male (5 years old Caucasian-American male, Figure 1, lane 5) and the remaining four were female (10 months old African-American female, Figure 1, lane 7; 5 years old Caucasian-American female, Figure 1, lane 9; 2 years old African-American female, Figure 1, lane 11; 4 years old Caucasian-American female, Figure 1, lane 22).

### Characterisation of mutation/deletion in 475 bp genomic regions

The cause for the multifold decreased intensity of 475 bp AP-PCR amplified locus in the tumour samples could be due to either mutation (s) at the primer-binding site(s) and/or due to deletion of this region in the genome of Wilms tumours.

**Figure 2:**
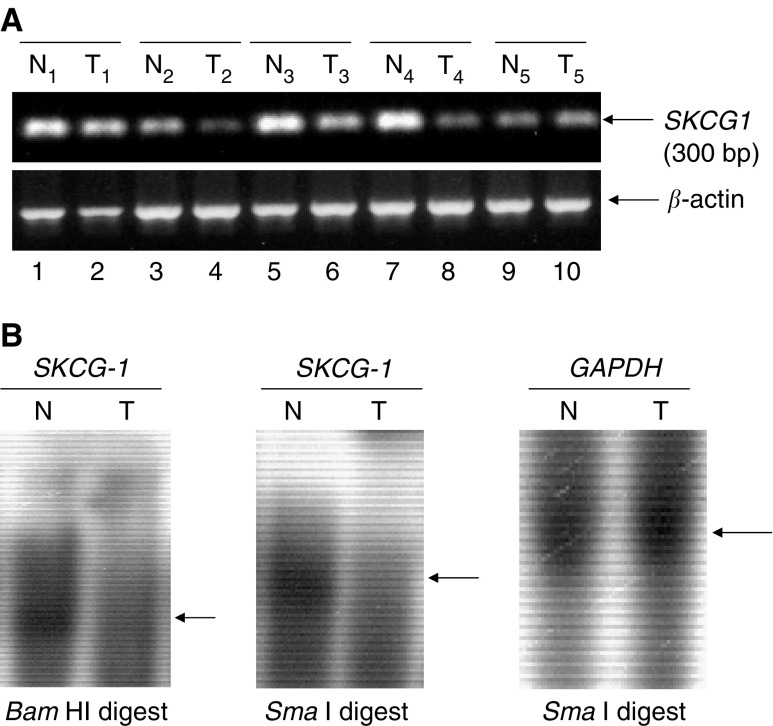
Sequence characterised amplified region (SCAR) (**A**) and Southern blot (**B**) analysis showing genomic loss of *SKCG-1* in Wilms tumours (T) and its presence in corresponding uninvolved normal (N) kidney tissue. The primers used for SCAR amplification are mentioned in Materials and Methods section. Restriction enzymes, *BamH1* and *Sma1* digested genomic DNA (10 *μ*gs) from Wilms tumours (T) and corresponding uninvolved (N) tissue was separated by electrophoresis, transferred to nylon membrane and hybridised with radioactive-labelled *SKCG-1*-or *GAPDH*-specific probe as mentioned in materials and methods. Arrow signs in figures indicate the hybridisation signal of *SKCG-1* in uninvolved normal tissue DNA and its loss in the tumour DNA sample. Control hybridisation with *GAPDH* (Glyceraldehyde-3-Phosphate Dehydrogenase) probe verified the equal amount of DNA loaded.

Sequence Characterised Amplified Region (SCAR) analysis was performed to investigate whether the loss of 475 bp AP-PCR amplified region is a result of allelic loss. Using SKCG*-1*- specific primer as mentioned in RT-PCR section of materials and methods, we amplified genomic sequence of *SKCG-1* from Wilms tumours and their corresponding uninvolved kidney tissue DNA. The combination of two *SCAR* primers generated a single PCR amplified fragment of expected size (300 bp); however, the intensity of this fragment was reduced to almost half in tumours as compared to their corresponding uninvolved tissue samples (Figure 2A). The tumour samples showing reduced intensity in *SCAR* analysis were the same samples that revealed reduced intensity in 475 bp AP-PCR amplified fragment (samples T_2_, T_3_, T_4_, T_5_ in Figures 1 and 2A). Thus, the result of SCAR analysis indicates loss of one allele of *SKCG-1* in the tumours. Matching normal tissue samples were uninvolved surrounding tissue of tumours. One of the normal (N5) tissues DNA consistently produced a profile similar to the matching tumour in both AP-PCR (Figure 1, lane 10) and SCAR amplification (Figure 2A, lane 9). This could be explained by the presence of similar genetic changes in tumour as well as in the surrounding pathologically normal tissue (N5). Though these cells have undergone the genetic changes, however, they have still not been morphologically changed. Thus, some of the pathologically normal looking cells may not be genetically normal.

We also performed Southern blot analysis with samples where enough DNA was available for this study. Presence of a hybridisation signal in uninvolved kidney tissue DNA and its absence in the corresponding tumour DNA was observed (Figure 2B). Thus, the result of Southern blot analysis confirmed the loss of *SKCG-1* genomic region in Wilms tumour tissue.

In order to identify mutations, if any, in the *SKCG-1* allele that is still present in the tumour, genomic sequence of *SKCG-1* (from base +1 to +1692) was amplified from DNA of tumour as well as from matching normal tissue and sequenced. Comparison of sequence from normal tissue DNA with corresponding tumour tissue DNA did not reveal any mutation in the *SKCG-1* allele in tumours.

### Determination of transcript size of SKCG-1 gene by Northern

**Figure 3:**
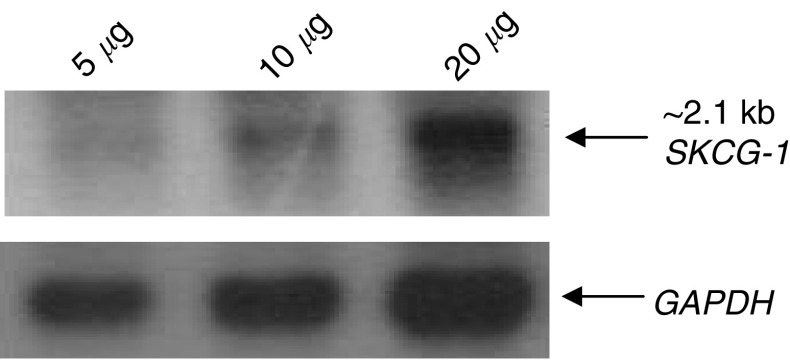
Northern blot analysis of *SKCG-1*. Total RNA isolated from immortalised embryonic kidney epithelial cells (HEK 293) was separated by electrophoresis, transferred to nylon membrane and hybridised with nonradioactive PCR generated-DIG-labelled *SKCG-1* gene-specific probe. Control hybridisation with *GAPDH* (Glyceraldehydes-3-phosphate dehydrogenase) probe verified the amount of RNA loaded.

To determine the size of the *SKCG-1* gene transcript, Northern analysis was performed with total RNA isolated from HEK293 cells derived from human embryonic kidney and using the PCR-generated probe as described in material and methods. A single band corresponding to the *SKCG-1* transcripts of ∼2.1 kb was observed (Figure 3).

### Isolation of full-length cDNA by RACE

**Figure 4:**
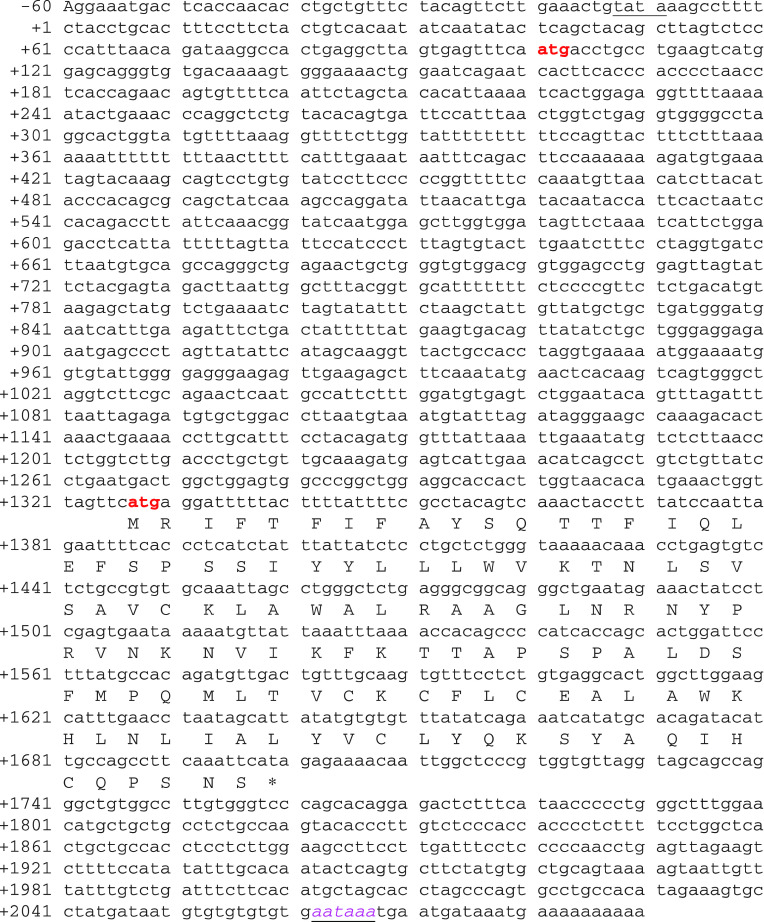
Full-length cDNA sequence and deduced amino-acid sequence of *SKCG-1*. The full-length *SKCG-1* cDNA encodes a predicted polypeptide of 124 amino acids. The polyadenylation signal is underlined. Sequence has been submitted to the NCBI database and the assigned GenBank accession number for *SKCG1* is AY662656.

The AP-PCR amplified product of 475 bp with primer OPA14 using DNA from uninvolved kidney tissue was gel eluted, reamplified, cloned and sequenced as described in material and methods. The final DNA sequence of 475 bp AP-PCR fragment (Figure 4, from +1089 to +1564) obtained from five different clones was compared with the known gene/genomic sequence in the GenBank. BLAST analysis revealed that there was a complete match (100% sequence similarity) of 475 bp sequences with Homosapiens chromosome 11 working draft sequence segment (NT_033899.7) and genomic clone (accession number AP003027) from human chromosome 11q as well as with one of the human expressed sequence tag (EST) (accession number AA935177). Interestingly, this EST has been reported from the cDNA library made from human kidney tumour tissue. By sequencing of this EST clone, we obtained 1.37 kb of *SKCG-1* (Figure 4, from +133 to +1503). Finally, 2.09 kb full-length cDNA sequence of *SKCG-1* was obtained by performing 5′ and 3′ rapid amplification of cDNA ends (RACE) using the 1.37 kb sequence information. 5′ RACE added 0.133 kb (Figure 4, from +1 to +133) whereas the remaining 0.587 kb sequence (Figure 4, from +1503 to + 2090) was obtained by 3′ RACE. The size of the 2.09 kb full-length transcript was very close to that of 2.1 kb as determined by northern. A comparison of the full-length cDNA sequence obtained by RACE with the genomic sequence revealed that this transcript is of single exon without any intervening intron sequence. Full-length cDNA sequence of *SKCG-1* has been submitted to the NCBI database and the assigned GenBank accession number for *SKCG-1* is AY662656.

### Distribution of SKCG-1 gene expression in various human tissues

**Figure 5:**
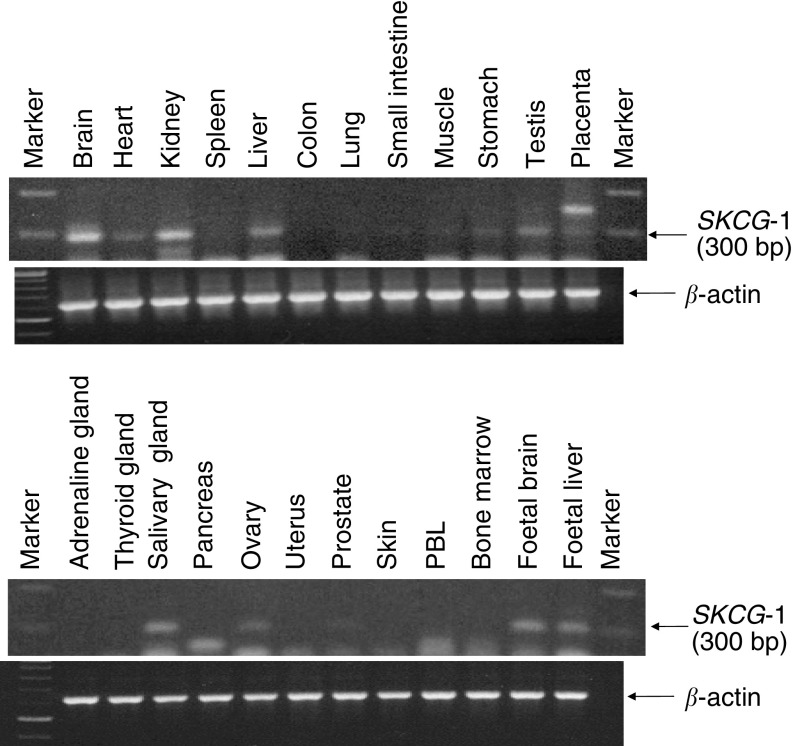
Distribution of *SKCG-1* gene expression in various human tissues. Expression of *SKCG-1* gene among the various tissues analysed by *SKCG-1* gene-specific PCR using tissue-specific cDNA panel (OriGene Technologies). Arrow indicates the expected size (300 bp) PCR product of *SKCG-1* from tissue-specific cDNA.

To determine the distribution of *SKCG-1* gene transcript in various human tissues, PCR amplification with *SKCG-1* gene-specific primer was performed on a panel of cDNA from various human tissues (Origene Inc). *SKCG-1* gene-specific amplification product of expected size (300 bp) was abundantly present in brain, kidney, liver, testis, salivary gland, foetal brain, foetal liver, whereas relatively lower expression of this gene transcript was found in heart, small intestine, muscle, stomach, placenta, and prostate (Figure 5). No expression of this gene transcript was detected in spleen, colon, lung, adrenal gland, pancreas, uterus, skin, PBL, and bone marrow (Figure 5). An amplification product of higher size (∼360 bp) in placenta and a lower size (∼270 bp) in pancreas was observed (Figure 5).

### Expression of SKCG-1 gene transcript in Wilms and breast tumours, and their corresponding uninvolved tissue

**Figure 6:**
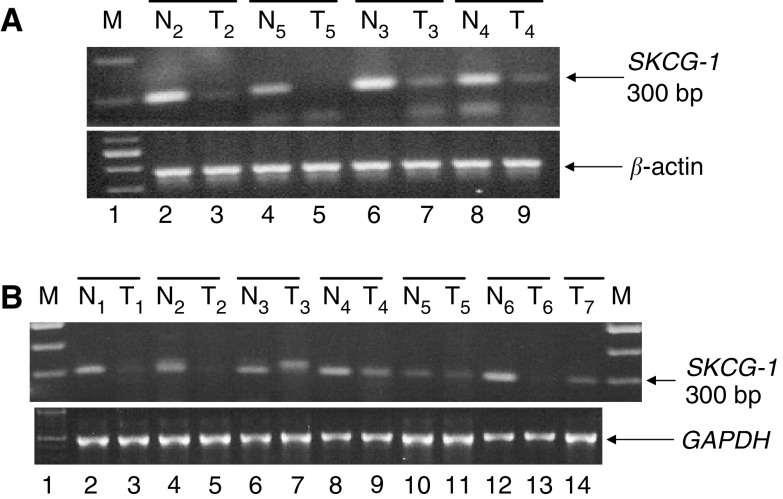
Semiquantitative RT–PCR analysis of *SKCG-1* transcript in human Wilms (**A**), and breast (**B**) tumours (T) and corresponding uninvolved normal (N) kidney tissue. Amplification of *β*-actin or *GAPDH* was used as control for similar amount of RNA taken for RT reaction.

We examined the expression pattern of *SKCG-1* gene transcripts by semi-quantitative RT–PCR analysis in Wilms (*N*=4) and breast (*N*=7) tumours *and their* corresponding uninvolved tissues. Expression of this gene was found to be either completely absent or hardly detectable in Wilms (Figure 6A) and breast (Figure 6B) tumours; however, its expression was readily detectable in their corresponding uninvolved tissues. These Wilms tumour samples were the same samples that revealed loss of 475 bp AP-PCR locus (T_2_, T_3_, T_4_, and T_5_, Figures 1 and 6A). An amplification product of higher size (320 bp) was observed in one of the breast tumour sample (Figure 6B, lane 7).

### Silencing of SKCG-1 by siRNA in HEK 293 cells

**Figure 7:**
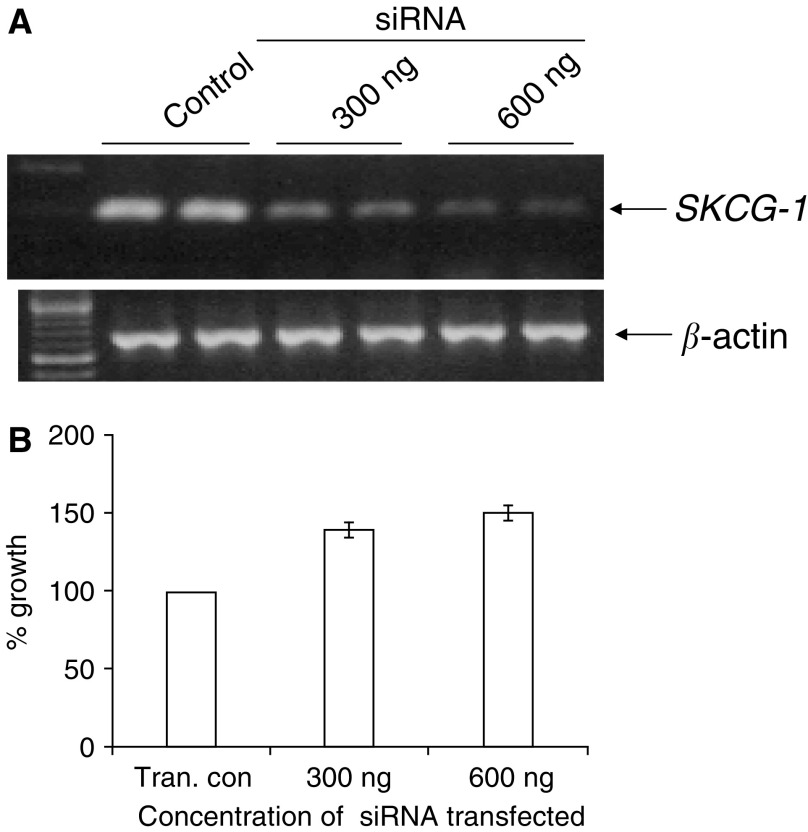
Effect of siRNA-mediated silencing of *SKCG-1* transcripts on the growth of human embryonic kidney epithelial cells. (**A**) *SKCG-1* gene-specific siRNA oligos was transfected into HEK293 cells. Cells treated in parallel with transfection reagent without siRNA oligos served as control in transfection experiment. After 48 h of transfection, the total RNA was isolated, RT was performed, and transcript level was detected by PCR using *SKCG-1* specific primers. Amplification of *β*-actin was used as control for similar amount of RNA taken for RT reaction. (**B**) Histogram showing the siRNA concentrations transfected and its effect on the growth of HEK 293 cells.

To determine the extent of gene silencing by siRNA, the total RNA was isolated from HEK293 cells at 48-h post-transfection and semiquantitative RT-PCR was performed. The *SKCG-1* gene transcript in siRNA-transfected cells was much lower as compared to the controls, indicating thereby the silencing of the gene (Figure 7A). To investigate whether the silencing of *SKCG-1* gene has any effect on the cell growth, we performed cell counts before isolating the RNA. The cell counts data revealed a 40% increase in the growth of human embryonic kidney cells as a result of *SKCG-1* gene silencing compared with the growth of control cells with constitutive expression of *SKCG-1* (Figure 7B).

### Chromosomal localisation of SKCG-1 by fluorescence *in situ* hybridisation (FISH)

**Figure 8:**
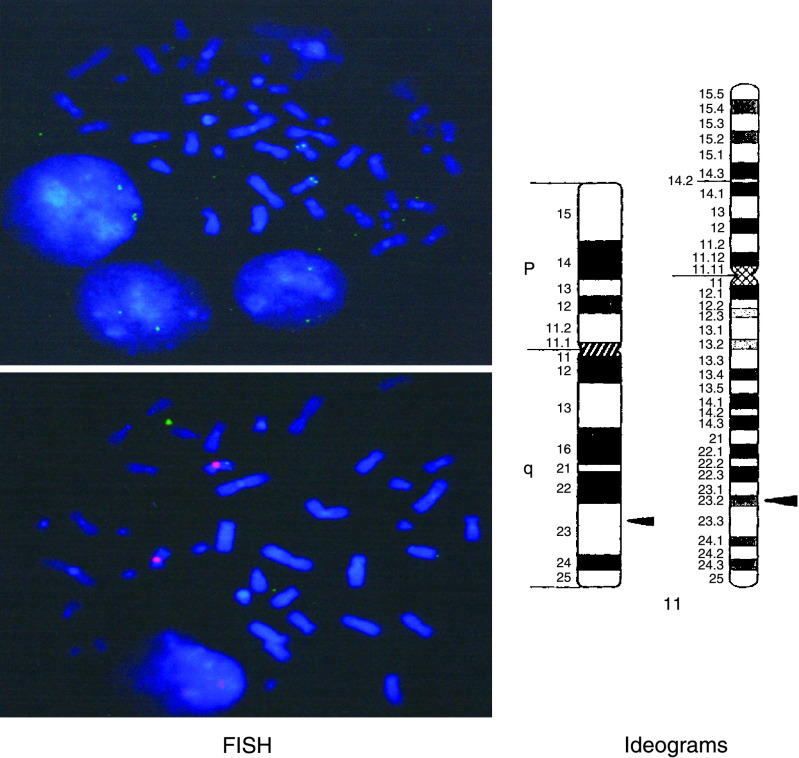
Chromosome localisation of *SKCG-1* ascertained by fluorescence *in situ* hybridisation analysis. *Left panel*: Representative metaphase is shown exhibiting specific hybridisation signal (green fluorescent) on the long arm of chromosome 11. A centromeric probe specific to the chromosome 11 was simultaneously hybridised (Red fluorescent). No signal was detected on any other chromosome using these probes. *Right panel*: Two ideograms illustrating the chromosomal position of *SKCG-1* at 11q23.2.

Initial experiment of FISH with human BAC clone containing 475 bp region of *SKCG-1* gene as a probe resulted in the specific hybridisation signal on the long arm of a group C chromosome that was believed to be chromosome 11 on the basis of size, morphology, and banding pattern. A second experiment with a biotin-labelled probe, which was specific for the centromere of chromosome 11, was cohybridised with *SKCG-1* BAC clone. This experiment resulted in the specific labelling of the centromere in red and long arm in green of chromosome 11. Measurement of 10 specifically labelled chromosome 11 demonstrated that *SKCG-1* BAC clone is located at a position, which is 73% of the distance from the centromere to the telomere of chromosome arm 11q, an area that corresponds to band 11q23.2 (Figure 8). A total of 80 metaphase cells were analysed with 75 exhibiting specific hybridisation signal.

### Structural analysis and coding potential of SKCG-1 gene

Analysis of 2 kb upstream genomic sequence of *SKCG-1* revealed a promoter region containing TATA box located at −13 bp upstream of transcription start site (underlined in Figure 4). Bioinformatic analysis using software program (www.ebi.ac.uk/servicestmp) revealed potential methylation sites (CpG lots) located between −600 to −900 bp in the promoter region of *SKCG-1*. This region corresponds to nucleotide 106503–106803 of human genomic clone, NCBI accession number AP003027. Sequence analysis of 2.09 kb full-length cDNA revealed two translation start sites (ATG sequence) present at +101 and +1327 bp (represented by bold letters in Figure 4), and a polyadenylation signal sequence (AATAAA) present at +2062 bp (represented by italics in Figure 4). ORF analysis revealed that the cDNA sequence of this gene has an open reading frame encoding a protein of 124 amino acids in frame +1 (Figure 4). Domain analysis using NCBI conserve domain database revealed that this gene does not have any conserved functional domain. The EMBL bioinformatic harvester program Website has analysed the detail structure of *SKCG-1* peptide. SOSUI software system has detected two transmembrane helices in the *SKCG-1* peptide. The primary transmembrane helices is represented by transmembrane region (N-terminal, AAno.24)-SIYYLLLWVKTNLSVSAVCKLAW- (C-terminal, AAno.46) and the secondary transmembrane helices is represented by transmembrane region (N-terminal, AAno.86)-VCKCFLCEALAWKHLNLIALYVC- (C-terminal, AAno.108) in *SKCG-1* peptide. On the basis of the presence of these two transmembrane helices, the *SKCG-1* has been predicted as a transmembrane protein. The predicted probability value of its localisation is 44.4% for extracellular including cell wall, 22.2% for cytoplasmic, 11.1% for golgi, 11.1% for nuclear, and 11.1% for endoplasmic reticulum. The detail structure of *SKCG-1* peptide, location of transmembrane region and its hydropathy profile as well as helical wheel diagram is available at the EMBL bioinformatic harvester website (http://harvester.embl.de/harvester/Q51SC8).

**Figure 9:**
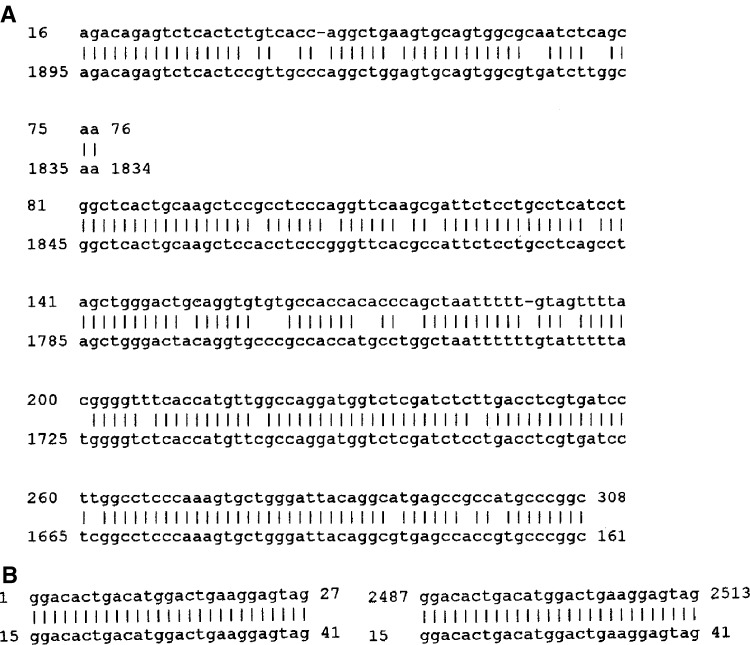
Sequence alignment at cDNA level of *SKCG-1* with a solute carrier family 14 (urea transporter) gene (**A**), and SOS2-like protein kinase mRNA (**B**).

No significant homology (over full-length cDNA of *SKCG-1*) of this gene with any known gene in NCBI GenBank database was found. However, sequence similarity search using database of Celera Genomics revealed that *SKCG-1* had 89% similarity over a 229-nucleotide stretch and 85% similarity over a 62-nucleotide stretch with a solute carrier family 14 (urea transporter) gene (Celera accession number NP_009094) reported from human kidney by Olives *et al* (1996) (Figure 9A). BLAST analysis revealed that a portion (from +497 to +522) of *SKCG-1* gene has significant homology (96% identity) with several cDNA clones, for example, NCBI accession numbers BU542594, BU632939, BU543849, BQ013859, BM993263, BF002848), indicating thereby that this region represents a conserved sequence across these genes. Another potentially important region found was a stretch of 27 nucleotides starting from +1 (5′-end) that revealed 100% sequence identity with SOS2-like Protein Kinase mRNA, accession number AF 525402.1 (Figure 9B).

## DISCUSSION

The most important finding of this study is the identification of an uncharacterised growth regulatory gene located at q23.2 region of chromosome 11. We observed genomic loss of this gene in 38% of the human Wilms tumour samples analysed. Several genes of pathogenic significance, for examples, *RCK, PLZP, LPC, RCK*, in haematopoietic neoplasm have been mapped to 11q23 (Stilgenbauer *et al*, 1996). However, no gene/gene mutations have been identified at 11q 23.2, and hence this is the first report of identification of a gene at this chromosomal region from Wilms tumour.

Our data indicate that the expression of *SKCG-1* gene at transcript level is either completely absent or undetectable in Wilms and breast tumour samples. Interestingly, those Wilms tumour samples that had genomic loss of *SKCG-1* also had reduced or loss of *SKCG-1* transcript (Figures 1 and 6). This coincidence of loss at DNA level, and loss of gene expression at transcript level within a tumour indicates that presumably reduced/loss of this gene expression is a consequence of either complete loss (homozygous deletion) as observed in Southern blot analysis, or loss of one allele and/or silencing of remaining one allele by mutations/promoter methylation. Sequence analysis of the allele that is still present in tumours did not reveal any mutation. Thus more than 50% reduction in the expression in some samples could be due to loss of one allele by deletion and silencing of the remaining allele possibly by methylation of *SKCG-1* promoter region. Presence of potential CpG lots in the *SKCG-1* promoter region further supports the probability of *SKCG-1* promoter region methylation as a causal factor for the silencing of expression of the allele that is still present in tumours. However, further study is needed to identify the exact cause for the loss of expression of *SKCG-1* allele that is still present in tumour.

We did not find any association of the observed genetic alteration with either specific histopathology, grade and stage of tumour or age as well as race of the patients in the samples used in this study. However, it seems that this genetic alteration is more frequent in female genetic background than in male as four out of five samples showing genetic alteration and loss of gene expression were from female patients. This gender-associated genetic alteration in *SKCG-1* remains to be validated by more number of samples.

Though we have not confirmed tumour suppressor activity of *SKCG-1*, however, the loss of expression of *SKCG-1* in highly proliferative human Wilms and breast tumours suggest that it is a potential growth regulatory gene. A 40% increase in the cell growth as a result of siRNA-mediated silencing of this gene in human embryonic kidney epithelial cells further strengthens the fact that *SKCG-1* controls the growth/proliferation of human kidney epithelial cells. The expression of *SKCG-1* in various tissue including kidney and its loss in Wilms and breast tumours indicates that this gene is not kidney specific and it may have a role in other tissue too. Whether the *SKCG-1* has similar growth regulatory function as observed in kidney tissue, and its loss of expression in their corresponding tumour tissue, remains to be investigated. The observed RT–PCR product of higher than the expected size in placenta and in one of the breast tissue samples, and lower than expected size in pancreas indicates that the spliced forms of *SKCG-1* also exits.

The most striking feature of the *SKCG-1* full-length cDNA sequence is the presence of very small open reading frames. The biggest open reading frame found in this gene was of 124 amino acids in frame +1. The alternative use of CTG or ACG as the initiation codon for translation (Kozak, 1996) does not significantly lengthen any of the open reading frames. On the basis of the presence of polyadenylated RNA molecule, several genes have been ruled out being as pseudogenes (Bussemakers *et al*, 1999). As the *SKCG-1* is expressed as polyadenylated RNA molecule with an ORF, and has function in controlling the cell growth as evident by siRNA silencing, the possibility of *SKCG-1* gene as a pseudogene can be ruled out.

Thus, in the perspective of the function of this gene, there are two logical explanations: (i) either it encodes for a very small functional protein or, (ii) it acts as a riboregulator. The evidence in favour of the first explanation that it encodes for a small functional protein is that it has DNA sequence similarity with urea transporter gene and also with protein kinase. The prediction of *SKCG-1* as a transmembrane protein based on the presence of two transmembrane helices in its peptide further strengthens the possibility of *SKCG-1* as urea transporter gene in the membrane. However, further study is needed to ascertain whether it encodes any protein and its function as a transporter located in the membrane.

The other possibility of this gene being a riboregulator cannot be ruled out as well. In the last few years an emerging group of mRNA-like noncoding RNAs have been discovered whose function and mechanisms of action remain poorly understood (Erdmann *et al*, 2001). This group of RNA lacks a protein coding capacity, and most probably they exert their action mainly or exclusively at the RNA level. These RNA molecules that function as genetic regulators are also known as ‘RNA riboregulator’. There are growing evidences which suggest that these riboregulators have important biological functions in development, differentiation, DNA damage, heat-shock responses, and tumorigenesis (Crespi *et al*, 1994; Velleca *et al*, 1994; Takeda *et al*, 1998). Several genes, for example, *H19, His-1, Bic*, that code for functional noncoding mRNAs have been found to play role in tumorigenesis (Askew and Xu, 1999). On the basis of our first report of identification and characterisation of *SKCG-1* gene, we propose that *SKCG-1* belongs to a distinct class of gene with potential function in kidney cell biology and in kidney tumorigenesis. In this report with several experiments we have provided the evidences for the role of *SKCG-1* in growth control of malignant and normal kidney epithelial cells as well as its loss of expression in Wilms and breast tumours. Thus, our results support the potential value of *SKCG-1* as new target for future clinical therapies to restore the normal cellular function of kidney and breast tissue.

## References

[bib1] Askew DS, Xu F (1999) New insights into the function of noncoding RNA and its potential role in disease pathogenesis. Histology Histopath 14(1): 235–241 10.14670/HH-14.235 9987668

[bib2] Bardeesy N, Falkoff D, Petruzzi MJ, Nowak N, Zabel B, Adam M, Aguiar MC, Grundy P, Shows T, Pelletier J (1994) Anaplastic Wilms tumour, a subtype displaying poor prognosis, harbors p53 gene mutations. Nat Genet 7(1): 91–97 8075648 10.1038/ng0594-91

[bib3] Bussemakers MG, Van Bokhoven A, Verhaegh GW, Smit FP, Karthaus HF, Schalken JA, Debruyne FM, Ru N, Isaacs WB (1999) DD3: A new prostate-specific gene highly overexpressed in prostate cancer. Cancer Res 59(23): 5975–5979 10606244

[bib4] Call KM, Glaser T, Ito CY, Buckler AJ, Pelletier J, Haber DA, Rose EA, Kral A, Yeger H, Lewis WH, Jones C, Houseman DE (1990) Isolation and characterisation of a zinc finger polypeptide gene at the human chromosome 11 Wilms tumour locus. Cell 60(3): 509–520 2154335 10.1016/0092-8674(90)90601-a

[bib5] Crespi MD, Jurkevitch E, Poiret M, d'Aubenton-Carafa Y, Petrovics G, Kondorosi E, Kondorosi A (1994) Enod 40, a gene expressed during nodule organogenesis, codes for a non-translatable RNA involved in plant growth. EMBO J 13(21): 5099–5112 7957074 10.1002/j.1460-2075.1994.tb06839.x PMC395456

[bib6] Erdmann VA, Barciszewska MZ, Szymanski M, Hochberg A, de Groot N, Barciszewski J (2001) The non-coding RNAs as riboregulators. Nucleic Acids Res 29(1): 189–193 11125087 10.1093/nar/29.1.189 PMC29806

[bib7] Francke U (1994) Digitized and differentially shaded human chromosome ideograms for genomic applications. Cytogenet Cell Genet 65(3): 206–218 8222762 10.1159/000133633

[bib8] Frohman MA (1993) Rapid amplification of complementary DNA ends for generation of full-length complementary DNAs: Thermal RACE. Meth Enzymol 218: 340–356 10.1016/0076-6879(93)18026-9 7685466

[bib9] Gessler M, Poustka A, Cavenee W, Neve RL, Orkin SH, Bruns GA (1990) Homozygous deletion in Wilms tumours of a zinc-finger gene identified by chromosome jumping. Nature 343(6260): 774–778 2154702 10.1038/343774a0

[bib10] Grundy PE, Telzerow PE, Breslow N, Moksness J, Huff V, Paterson MC (1994) Loss of heterozygosity for chromosomes 16q and 1p in Wilms' tumours predicts an adverse outcome. Cancer Res 54: 2331–2333 8162576

[bib11] Hing S, Lu YJ, Summersgill B, King-Underwood L, Nicholson J, Grundy P, Grundy R, Gessler M, Shipley J, Pritchard-Jones K (2001) Gain of 1q is associated with adverse outcome in favorable histology Wilms' tumours. Am J Pathol 158: 393–398 11159177 10.1016/S0002-9440(10)63982-X PMC1850292

[bib12] Koufos A, Grundy P, Morgan K, Aleck KA, Hadro T, Lampkin BC, Kalbakji A, Cavenee WK (1989) Familial Wiedemann-Beckwith syndrome and a second Wilms tumour locus both map to 11p15.5. Am J Human Genet 44(5): 711–719 2539717 PMC1715635

[bib13] Kozak M (1996) Interpreting cDNA sequences: some insights from studies on translation. Mammalian Genome 7(8): 563–574 8679005 10.1007/s003359900171

[bib14] Maniatis T, Fritsch EF, Sambrook J (1982) Molecular Cloning: A laboratory Manual. Cold Spring Harbor, New York

[bib15] Mannens M, Slater RM, Heyting C, Bliek J, de Kraker J, Coad N, de Pagter-Holthuizen P, Pearson PL (1988) Molecular nature of genetic changes resulting in loss of heterozygosity of chromosome 11 in Wilms tumours. Human Genet 81(1): 41–48 2848758 10.1007/BF00283727

[bib16] Matsunaga E (1981) Genetics of Wilms tumour. Hum Genet 57: 231–246 6265341 10.1007/BF00278936

[bib17] Maw MA, Grundy PE, Millow LJ, Eccles MR, Dunn RS, Smith PJ, Feinberg AP, Law DJ, Paterson MC, Telzerow PE (1992) A third Wilms tumour locus on chromosome 16q. Cancer Res 52(11): 3094–3098 1317258

[bib18] Olives B, Martial S, Mattei MG, Matassi G, Rouss RP, Cartron JP, Bailly P (1996) Molecular characterization of a new transporter in kidney. FEBS Lett 286(2-3): 156–160 10.1016/0014-5793(96)00425-5 8647271

[bib19] Radice P, Perotti D, De Benedetti V, Mondini P, Radice MT, Pilotti S, Luksch R, Fossati Bellani F, Pierotti MA (1995) Allelotyping in Wilms tumours identifies a putative third tumour suppressor gene on chromosome 11. Genomics 27(3): 497–501 7558032 10.1006/geno.1995.1082

[bib20] Singh KP, Roy D (1999) Detection of mutation(s) or polymorphic loci in the genome of experimental animal and human cancer tissues by RAPD/AP-PCR depend on DNA polymerase. Int J Oncol 14(4): 753–758 10087325 10.3892/ijo.14.4.753

[bib21] Singh KP, Roy D (2001) Identification of novel breast tumour-specific mutation(s) in the q11.2 region of chromosome 17 by RAPD/AP-PCR fingerprinting. Gene 269(1–2): 33–43 11376935 10.1016/s0378-1119(01)00458-9

[bib22] Stokke T, Collins C, Kuo WL, Kowbel D, Shadravan F, Tanner M, Lallioniemi A, Kallioniemi OP, Pinkel D, Deaven L (1995) A physical map of chromosome 20 established using fluorescence *in situ* hybridization and digital image analysis. Genomics 26(1): 134–137 7782072 10.1016/0888-7543(95)80092-z

[bib23] Stilgenbauer S, Liebisch P, James MR, Schroder M, Schlegelberger B, Fischer K, Bentz M, Lichter P, Dohner H (1996) Molecular cytogenetic delineation of a novel critical genomic region in chromosome bands 11q22.3–23.1 in lymphoproliferative disorders. Proc Natl Acad Sci USA 93(21): 11837–11841 8876224 10.1073/pnas.93.21.11837 PMC38145

[bib24] Takeda K, Ichijo H, Fujii M, Mochida Y, Saitoh M, Nishitoh H, Sampath TK, Miyazono K (1998) Identification of a novel bone morphogenic protein-responsive gene that may function as a noncoding RNA. J Biol Chem 273(27): 17079–17085 9642273 10.1074/jbc.273.27.17079

[bib25] Velleca MA, Wallace MC, Merlie JP (1994) A novel synapse-associated noncoding RNA. Mol Cell Biol 14(11): 7095–7104 7523860 10.1128/mcb.14.11.7095-7104.1994 PMC359243

[bib26] Volkher S, Alex J, Van der E, Jochemsen AG (2001) WT1 proteins: functions in growth and differentiations. Gene 273: 141–161 11595161 10.1016/s0378-1119(01)00593-5

